# Why Do Women Deliver at Home? Multilevel Modeling of Ethiopian National Demographic and Health Survey Data

**DOI:** 10.1371/journal.pone.0124718

**Published:** 2015-04-15

**Authors:** Henock Yebyo, Mussie Alemayehu, Alemayehu Kahsay

**Affiliations:** Department of Public Health, College of Health Sciences, Mekelle University, Mekelle, Ethiopia; Université Catholique de Louvain, BELGIUM

## Abstract

**Background:**

Despite of the existing intensive efforts to improve maternal health in Ethiopia, the proportion of birth delivered at home remains high and is still the top priority among the national health threats.

**Objective:**

The study aimed to examine effects of individual women and community-level factors of women’s decision on place of delivery in Ethiopia.

**Methods:**

Data were obtained from the nationally representative 2011 Ethiopian Demographic and Health Survey (EDHS) which used a two-stage cluster sampling design with rural-urban and regions as strata. The EDHS collected data from a big sample size but our study focused on a sample of 7,908 women whose most recent birth was within five years preceding 2011 and 576 communities in which the women were living in. The data were analyzed using a two-level mixed-effects logistic regression to determine fixed-effects of individual- and community-level factors and random-intercept of between-cluster characteristics.

**Results:**

In the current study, 6980 out of 7908 deliveries (88.3%) took place at home. Lower educational levels (OR=2.74, 95%CI:1.84,4.70; p<0.0001), making no or only a limited number of ANC visits (OR=3.72,95%CI:2.85, 4.83; p<0.0001), non-exposure to media (OR=1.51, 95%CI 1.13, 2.01; p=0.004), higher parity (OR=2.68, 95%CI:1.96,3.68; p<0.0001), and perceived distance problem to reach health facilities (OR=1.29, 95%CI:1.03,1.62; p=0.022) were positively associated with home delivery. About 75% of the total variance in the odds of giving birth at home was accounted for the between-community differences of characteristics (ICC=0.75, p<0.0001). With regard to community-level characteristics, rural communities (OR=4.67, 95%CI:3.06,7.11; p<0.0001), pastoralist communities (OR=4.53, 95%CI:2.81,7.28; p<0.0001), communities with higher poverty levels (OR=1.49 95%CI:1.08,2.22; p=0.048), with lower levels of ANC utilization (OR=2.01, 95%CI:1.42,2.85; p<0.0001) and problem of distance to a health facility (OR=1.29, 95%CI:1.03,1.62; p=0.004) had a positive influence on women to give birth at home.

**Conclusions:**

Not only individual characteristics of women, but also community-level factors determine women’s decision to deliver at home.

## Introduction

Globally, Maternal Mortality Ratio (MMR) has fallen by 45% between 1990 and 2013. There were an estimated 289,000 maternal deaths in 2013, yielding a MMR of 210 per 100,000 live births [1]. Developing countries accounted for 99% of global maternal deaths; Sub-Saharan Africa (SSA) alone accounted for 62% of them. Ethiopia is one of the ten countries that comprised 58% of the global maternal deaths [1]. In the latest report of Ethiopia Demography and Health Survey (EDHS) 2011, the MMR was estimated at 676/100,000 live births [2]. This is very distal to achieve the Millennium Development Goals target set at 267/100,000 live births [3]. Several reasons are accountable for the high MMR in SSA including Ethiopia. The lack of decision-making power of women within the family remains a challenge in many SSA countries [4,5]. Moreover, low levels of female education and attaining low care during pregnancy prevent women from seeking care, and accessing the best choices for themselves and their children’s health, resulting in critical delays, and unnecessary complications and deaths [4–7].

Studies in some countries have reported home deliveries ranging from 22% in Senegal to 65% in Tanzania and to 87.7% in Bangladesh [8–10]. The EDHS 2011 shows that home delivery comprises 90% of all deliveries in the country, regardless of expansion of strategies to increase service utilization and to improve maternal and child health [11]. Small studies in different regions of the country also support this figure. Similar to studies conducted in Bangladesh and Nigeria, small studies in Ethiopia show that the most frequent reasons for high rates of home delivery are distance to health facility, problems in transportation, lack of decision making power among women, low levels of antenatal care (ANC), and poor educational attainment [12–16]. To overcome the variability of these findings, however, providing representative evidences using robust methods is required to identify the effects of individual and community-level factors on home delivery in Ethiopia.

We hypothesized that not only individual women’s characteristics but also community characteristics and composition would influence women’s decision on place of delivery. Some studies have attempted to solve this issue [12–14,17,18]; but, instead have focused only on effects on individual women characteristics. This study, therefore, aimed to examine the effects of individual and community-level characteristics and between-community differences on women’s decision to deliver at home using multilevel modeling based on the EDHS 2011 data. The finding of this study would present better evidence for the policy makers and stakeholders, which in turn, may help designing and implementing appropriate interventions at different levels to decrease home delivery and for the betterment of women health, in general.

## Methods

### Demography and Health Survey design and population

**Fig 1:**
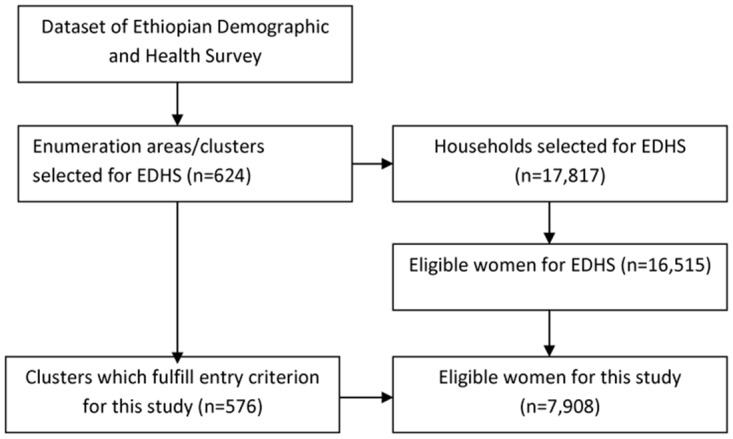
The flowchart for the data extraction procedure from EDHS 2011, Ethiopia.

We used data from the EDHS 2011, particularly data on individual women. Ethiopia Demography and Health Survey used a two-stage cluster sampling design with rural-urban and regions as strata. In EDHS 2011, a sample of 624 clusters was drawn by the Ethiopian Central Statistical Agency from its master sampling frame of census 2007. Cluster (community) was defined as a randomly selected area, which contained 150–200 households. In total, 17,817 households and 16,515 reproductive women age 15–49 were sampled using random selection from these clusters (Fig 1).

With respect to structure of the data, women are nested within household and household are nested within clusters. The survey was conducted from December 27, 2010 to June 3, 2010 in all the nine regions and two administrative councils of Ethiopia.

### Sample size

We included individual data of 7,908 women (weighted) whose last birth was alive and delivered within five years preceding 2011 and community characteristics of 576 clusters (weighted). For mothers with more than one births, we used the most recent birth for the study (Fig 1).

### Data source and definition of covariates

The DHS captures a wide scope of data, generally concerning the health of women, men and children. However, for the current study, we used specific data, related with maternal health. The primary entry criterion to this study was women having a live birth within five years preceding the DHS. For the analysis, we included individual variables of socioeconomic and demographic characteristics, obstetric, fertility, perception of women to access health facilities, access to health facility, mass media, and others. The outcome of interest for the study was place of delivery and was grouped into two categories: home and facility based delivery. Home delivery was defined as any birth that had taken place in the women’s or others’ home; while deliveries that occurred in governmental health post, health center, hospital and private clinic and hospital and NG health facilities were grouped as facility-based delivery. In Ethiopia if a birth takes place at home, it is unlikely skilled health professionals assist it. In this context, no home delivery in the EDHS survey was assisted by a nurse, doctor or midwife.

The study also focused on community characteristics. We took place of residence as urban versus rural without changing the original coding in the DHS dataset. However, the regions in Ethiopia are divided into eleven for administrative purpose; but, the delineation of the regions may not necessarily be related to the health status of their population. For this study, we have classified the regions into three contextual—agrarian, pastoralist and city dwellers—based on the characteristics of their population in relation to maternal health, particularly place of delivery. Based on their living ways, Ministry of Health of Ethiopia has clearly identified which regions are agrarian, pastoralist or city dwellers so as to make a contextual intervention for each region [19].

Except place of residence and geographic regions, the EDHS did not capture variables that can describe the characteristics of the communities. Yet, we created more community characteristics by aggregating the individual mothers’ characteristics within their clusters. The aggregates for clusters were computed using mean of the proportions of women in each category of a given variable. We categorized the aggregate of a cluster into groups based on the National Median Values. We used median since all distributions of the aggregates were not normally distributed. For the community ANC utilization, for example, we computed the proportion of ANC utilization in each cluster. Finally, we categorized these aggregate values into lower and higher ANC utilization based on the National median of ANC utilization. Ultimately, we used these individual and community level factors to answer why Ethiopian women still deliver at home.

### Data Analyses

#### Description of individual and community characteristics

The data were analyzed using STATA 11 (Stata Corporation, College Station, TX, USA). The different characteristics of women and communities were described using descriptive statistics. The proportions and frequencies were estimated after applying sample weights to the data to adjust for disproportionate sampling and non-responses.

#### Multivariable multilevel regression analysis

Since DHS data are hierarchical, i.e. mothers are nested within households, and households are nested within clusters, use of flat models could underestimate standard errors of the effect sizes, which consequently can affect decision on null hypothesis. In such data, mothers within same cluster may be more similar to each other than mothers in the rest of the country. This violates the assumption of flat models—independence of observations and equal variance across the clusters. Thus, we used two-level mixed-effects logistic regression model to test the effect sizes of individual and community factors on women’s decision to place of delivery and estimate the between-cluster variability of odds of home delivery. We ran four models: Empty model, model containing only individual factors, model containing community—level factors and model combining both individual and community-level factors. We fit the data into the model:

log[πij1−πij]=β0+β1X1ij+...+βnXnij+uoj+eij

Where

- *π*_*ij*_ is probability of the presence of home delivery
- *(1-π*_*ij*_) is probability of institutional delivery
- *β*_*0*_ is log odds of the intercept
- *β*_*1*_, *… β*_*n*_ are effect sizes of individual and community-level factors
- *X*_*1ij*_. . . *X*_*nij*_ are independent variables of individuals and communities
- The quantities *u*_Oj_ is random errors at cluster levels
- e_ij_ random errors at the individual levels

The distribution of *u*_*0j*_ is normal with mean 0 and variance σ^2^_u0_. The Intra-Class Correlation (ICC) was calculated using between-cluster variance and within cluster variance [*ICC* = *σu* ^2^ /(*σu* ^2^ + *π* ^2^ /3)]. In log distribution, the residual variance of women within a cluster is zero but variance is considered constant at π^2^/3. This helped to show the level of between-cluster correlation within a model and to compare the successive models by looking at the decline of the ICC. The Proportional Change in Variance (PCV) was also computed for each model with respect to the empty model to show how much of variability on the odds of home delivery be explained by the successive models. The PCV was calculated as *PCV* = (*V*_*e*_—*V*_*mi*_)/*V*_*e*_ where V_e_ is variance in women’s decision in the empty model and V_mi_ is variance in successive models. We used Variance Inflator Factor (VIF) to scrutiny high multicollinearity among the explanatory variables. The fixed effect sizes of individual and community-level factors on place of delivery were expressed using the Odds Ratio (OR) and the population effect sizes were estimated using 95% Confidence Interval (CI).

### Ethics statement

We accessed the data from MEASURE DHS database at http://dhsprogram.com/data/available-datasets.cfm. We retrieved data of women only. As the data were obtained from records, we could not consent women for accessing their records. However, the records were anonymized and de-identified prior to analysis. MEASUSRE DHS governs the DHS data of all countries and researchers can use the data obeying the data sharing policy. The organization accessed us the data after reviewing our proposal. We accepted the terms and conditions attached to data sharing policy; i.e, we need to keep the data confidential and we would not use the data for purposes other than the current study.

## Results

### Background characteristics of individual women

A large proportion of women in Ethiopia had no formal education (66.6%). Only 4.6% of the women had attended secondary school or higher. Similarly, nearly half (45.3%) of the women were unemployed—they were limited to household activities. In contrast, almost all (98.7%) of their husbands had jobs ranging from unskilled manual labor to professional. Estimated using the Principal Component Analysis (PCA) over a household’s ownership of selected assets, the household’s cumulative living standard was expressed by five quintiles. As such, the proportion of women was nearly equal among the wealth quintiles from 17.1% in richest to 22% in the poorest (Table 1).

**Table 1:** Background characteristics of women who had a live birth in the five years preceding the EDHS 2011, Ethiopia (n = 7,908).

Individual characteristics	n(%)
**Woman’s educational status**	
No education	5270(66.6)
Primary	2270(28.7)
Secondary	225(2.8)
Higher	142(1.8)
**Employment status of woman**	
No	3585(45.3)
Yes	4323(54.6)
**Perceived distance to a health facility to get medical help**	
Not a big problem	2142(27.1)
Big problem	5757(72.9)
**Preference for a female health provider**	
Not a big problem	1783(22.5)
Big problem	6125(77.4)
**Birth order of the last birth**	
1^st^	1399(17.7)
2^nd^ or 3^rd^	2462(31.1)
4^th^ or 5^th^	1814(22.9)
6^th^ +	2233(28.2)
**Number of ANC visits**	
None	4543(57.4)
1–3	1856(23.4)
4+	1508(19.1)
**Exposure to mass-media**	
Did not listened to/watched radio/TV	3217(40.7)
Either listened to radio or watched TV	2599(32.9)
Listened to/watched both radio and TV	2091(26.4)
**Woman’s empowerment based on participation in major decisions on household issues**	
No involvement	677(9.5)
Involved in one decision	723(10.1)
Involved in two decisions	904(12.6)
Involved in three decisions	1412(19.7)
Involved in four decisions	3446(48.1)

A woman is empowered if she made a decision on any of the household issues alone or together with her partner.

With regards to media exposure, 40.6% of women had not listened to radio or watched television in the course of a week. The DHS included questions to assess women’s empowerment in terms of involvement in all four household decisions mentioned during the survey either alone or with their husband. Ten percent of the women were not involved in any of the decisions, while the rest participated in at least one decision. Five in every ten of women were involved either in all four household decisions asked on the survey or together with their husband (Table 1).

In addition to the number of ANC visits, the quality of ANC can also be measured by the qualifications of the service providers. Nearly 42% of the women had attended ANC at least once from any provider including community health workers, while only 33.8% received ANC from a doctor, nurse or midwife which is supposed to be quality ANC service (Table 1).

### Background characteristics of communities

The unit of analysis for the characteristics of community-level factors was the cluster. For this study, we included 576 clusters in which all the women whose most recent birth was within five years preceding the EDHS 2011 were living in. Three-fourth of the clusters was sampled from rural areas. The different regions were contextually categorized into three groups based on their living settings. As such, 91.5% of the clusters were obtained from the agrarian regions, while the rest were from pastoralist regions followed by cities. The current study attempted to form community-level factors, by aggregating values of different individual characteristics. Accordingly, 55% of the clusters had higher poverty levels and 57% of them had lower ANC utilization levels based on the aggregate values derived from wealth index and ANC utilization of individual women, respectively (Table 2).

**Table 2:** The characteristics of clusters in Ethiopia Demography and Health Survey 2011, Ethiopia (n = 576).

Community characteristics	Category	n(%)
**Contextual regions**		
	Agrarian	526(91.2)
	Pastoralist	18(3.2)
	City dwellers	32(5.6)
**Place of residence**		
	Urban	135(23.5)
	Rural	441(76.5)
**Community distance to a health facility to get medical service**		
	Not a big problem	258(44.8)
	Big problem	318(55.2)
**Community poverty status**		
	Higher	316(54.9)
	Lower	260(45.1)
**Female educational attainment of communities**		
	Lower	292(50.6)
	Higher	284(49.4)
**Community media exposure**		
	Lower	284(49.2)
	Higher	292(50.8)
**Community ANC utilization**		
	Lower	330(57.3)
	Higher	246(42.7)
**Women’s empowerment in communities**		
	Lower	263(45.6)
	Higher	313(54.4)
**Perceived distance of a community to a health facility to get medical service**		
	Not a big problem^a^	258(44.8)
	Big problem^b^	318(55.2)

^a^Big problem: a cluster where at least half of the women say distance is a big problem to reach health facility.

^b^Not a big problem: a cluster where less than half of the women say that distance is a big problem to reach health facility.

### Place of delivery

In our study, 6,980(88.3%) of mothers delivered their most recent birth at home. Only 928(11.7%) of the women delivered their most recent birth in a health institution—governmental, private, and non-governmental health institutions.

### Multivariable multilevel analyses

A two-level mixed effects logistic regression model was used to analyze the effects of women’s individual characteristics and community-level factors in women’s decision on place of delivery. As depicted in the empty model, 75% of the total variance in the odds of giving birth at home was accounted for by between-cluster variation of characteristics (ICC = 0.75, p<0.0001). The between-cluster variability declined over successive models, from 75% in the empty model to 25% in individual-level only model, 21% in community-level only model, and 17% in the combined model. Accordingly, the combined model of individual-level, and community-level factors was selected for predicting women’s decision about place of delivery.

### Effect of individual women characteristics on place of delivery

The details of effect sizes of both individual and community-level factors on odds of home delivery are depicted in Tables 3, 4 and 5. Both educational-level of women and of their partners were independently and significantly associated with women’s decision to place of delivery. After adjusting for individual and community-level factors, the odds of giving birth at home was 2.7 times (OR 2.74; 95% CI 1.84, 4.70; p<0.0001), and 2 times (OR 2.05; 95% CI 1.44, 2.94; p<0.0001) higher among women with no education compared with those who attended secondary (or higher) school and primary school, respectively. Similarly, women partnered with uneducated husbands had 2 times higher odds of giving birth at home (OR 2.31; 95% CI 1.68, 3.18; p<0.0001) (Table 3).

**Table 3:** Predictors of women’s decision to place of delivery among women with a live birth five years preceding the 2011 EDHS, Ethiopia.

	Model	Individual factors	Community factors	Individual & community factors
Individual and community characteristics	Empty	OR (95% CI)	OR (95% CI)	OR (95% CI)
**Educational level of woman**				
No education		2.90 [1.90, 4.40]***		2.74 [1.84, 4.70] ***
Primary		2.03 [1.39, 2.97] ***		2.06 [1.44, 2.94] ***
Secondary or higher		1.00		1.00
**Educational level of partner**				
No education		2.28 [1.61, 3.22] ***		2.31 [1.68, 3.18] ***
Primary		1.79 [1.33, 2.41] ***		1.86 [1.42, 2.44] ***
Secondary or higher		1.00		1.00
**Wealth index quintile**				
Poorest		8.92 [6.01, 13.25] ***		1.15 [0.72, 1.83]
Poorer		9.38 [6.17, 14.26] ***		1.31 [0.82, 2.10]
Middle		9.71 [6.44, 14.65] ***		1.73 [1.09, 2.75]*
Rich		5.65 [4.06, 7.89] ***		1.38 [0.97, 1.98]
Richest		1.00		
**Media exposure**				
No exposure to TV/radio		1.47 [1.10, 2.00]		1.51 [1.13, 2.01]**
Exposure to either TV/ radio		1.37 [1.07, 1.77]		1.29 [1.02, 1.61]*
Exposure to both TV and radio		1.00		1.00
**Perceived problem of transport to get medical help**				
Big problem		1.13 [0.84, 1.53]		0.97 [0.71, 1.25]
Not a big problem		1.00		1.00
**Perceived distance to a health facility to get medical help**				
Big problem		1.78 [1.32, 2.38] ***		1.29 [1.03, 1.62]*
Not a big problem		1.00		1.00

**Table 4:** Predictors of women’s decision to place of delivery among women with a live birth five years preceding the 2011 EDHS, Ethiopia.

	Model	Individual factors	Community factors	Individual & community factors
Individual and community characteristics	Empty	OR (95% CI)	OR (95% CI)	OR (95% CI)
**Perception towards preference for female health provider**				
Not a big problem		1.00		
Big problem		1.01 [0.80, 1.27]		
**Birth order of the last birth**				
1^st^		1.00		1.00
2^nd^ and 3^rd^		1.85 [1.42, 2.41] ***		1.95 [1.53, 2.49] ***
4^th^ and 5^th^		2.80 [2.03, 3.85] ***		2.44 [1.81, 3.28] ***
6^th^ +		3.08 [2.21, 4.30] ***		2.68 [1.96, 3.68] ***
**ANC visits** None		5.10 [1.43, 2.41] ***		3.72 [2.85, 4.83] ***
1–3		2.03 [1.57, 2.61] ***		1.60 [1.26, 2.03] ***
4+		1.00		1.00
**Woman’s empowerment by number of major decisions**				
No any major decisions		1.12 [0.73, 1.70]		
Made one major decision		1.19 [0.81, 1.75]		
Made two major decisions		1.14 [0.81, 1.61]		
Made three major decisions		1.07 [0.82, 1.39]		
Major four major decisions		1.00		
**Contextual region**				
Agrarian			3.52 [2.53, 4.88] ***	3.22 [2.30, 4.49] ***
Pastoralist			6.26 [3.85, 10.20] ***	4.53 [2.81, 7.28] ***
City dwellers			1.00	1.00

**Table 5:** Predictors of women’s decision to place of delivery among women with a live birth five years preceding the 2011 EDHS, Ethiopia.

	Model	Individual factors	Community factors	Individual & community factors
Individual and community characteristics	Empty	OR (95% CI)	OR (95% CI)	OR (95% CI)
**Place of residence**				
Urban			1.00	1.00
Rural			7.73 [5.16, 11.58] ***	4.27 [2.80, 6.50] ***
**Community distance to heath facility**				
Big problem			2.11 [1.51, 2.95] ***	1.63 [1.16, 2·29]**
Not a big problem			1.00	1.00
**Community poverty status**				
Higher poverty			1.52 [1.04, 2.20]*	1.49 [1.08, 2.22]*
Lower poverty			1.00	1.00
**Female educational attainment of community**				
Higher			1.00	1.00
Lower			1.94 [1.34, 2.79] ***	1.20 [0.83, 1.73]
**Community ANC utilization**				
Higher			1.00	1.00
Lower			2.82 [1.98, 3.99] ***	1.90 [1.34, 2.60] ***
**Community media exposure**				
Higher			1.00	1.00
Lower			0.90 [0.63, 1.30]	0.73 [0.50, 1.05]
**Community women’s empowerment**				
Higher			1.00	1.00
Lower			1.12 [0.86, 1.54]	1.13 [0.84, 1.51]
**Random effects**				
ICC	0.75	0.25	0.21	0.17
PCV	N/A	88.7%	91.0%	93.0%

*p<0.05;

**p<0.01;

***p<0.0001.

Note: Tables 3, 4 and 5 contain predictors fitted simultaneously into one model. They are separately reported as a production requirement for publication.

Parity was highly significant to influence women’s decision about place of delivery. Controlling for the effect of individual and community factors such as education, frequency of ANC, media exposure, wealth index, community ANC utilization, and place of residence, multiparous women had higher odds of having home delivery than uniparous women. As birth order rose, women were increasingly likely to have home delivery. The odds of staying at home for giving birth among women with six or more births, 4–5 births, 2–3 births were 2.7 times (OR 2.68; 95% CI 1.96, 3.68; p<0.0001), 2.5 times (OR 2.44, 95% CI 1.81, 3.28; p<0.0001), and 2 times (OR 1.95, 95% CI 1.53, 2.49; p<0.0001) higher, respectively, than among uniparous women. Frequency of ANC was a statistically significant factor to affect women’s decision on place of delivery. The net odds of home birth among mothers with no history of ANC visits for their recent pregnancy was 3.7 times (OR 3.72; 95% CI 2.85, 4.83; p<0.0001) higher than among mothers who had four or more ANC visits. Even mothers who made 1–3 ANC visits had higher odds of delivery at home than mothers with the recommended four or more visits (Table 4).

To examine the influence that health promotion in the mass media would have encouraging institutional delivery, the study analyzed the effect of exposure to television or radio on women’s decision on place of delivery. Thus, non-exposure to mass media influenced women to deliver at home. Having no exposure to radio or TV messages was associated with 51% (OR 1.51; 95% CI 1.13, 2.01; p = 0.004) more odds of giving birth at home compared with mothers who watched television or listened to radio at least once a week. Even limited exposure to either radio or television had a positive effect on institutional delivery compare with non-exposure. In addition, the perceived problem of distance to get any medical service deterred mothers from delivering in a health facility (OR 1.29; 95% CI 1.02, 1.61; p = 0.047) (Table 3).

### Effect of community-level characteristics on place of delivery

The study aimed to show if the characteristics of the clusters in which women lived would have an effect on their decision about place of delivery, regardless of women’s individual characteristics. These results are net effects after controlling for the contribution of all the individual and community-level attributes. Related to place of residence, rural clusters had more odds to have a higher proportion of women giving birth at home (OR 4.67, 95% CI 3.06, 7.11; p<0.0001). Residence in pastoralist community had a strong positive net effect on home delivery, which exceeded by four-and-a-half times (OR 4.53, 95% CI 2.81, 7.28; p<0.0001) the level among city community. Similarly, women living in agrarian communities had greater odds (OR 3.22, 95% CI 2.30, 4.49; p<0.0001) of giving birth at home compared with women in city communities.

On this basis, more odds (OR 1.63; 95% CI 1.16, 2.29; p = 0.004) was appended with clusters (communities) where distance was a big obstacle to seeking medical services to prefer home birth. With regard to the cumulative average poverty status of clusters, women in clusters with higher relative poverty levels had greater likelihood of giving birth at home (OR 1.49; 95% CI 1.08, 2.22; p = 0.048) versus women in clusters with lower poverty levels.

Not only ANC utilization of individual women would have an effect on themselves to delivery at health facility, but also higher proportion of ANC utilization of community may influence the rest of women in the same community positively to deliver at health institutions or vise versa. Women’s decision to delivery at home was positively associative with communities having lower levels of ANC utilization. After controlling for all other characteristics, the result indicated that residence in a community with a lower level of women’s ANC utilization had 2 times (OR 2.01, 95% CI 1.42, 2.85; p<0.0001) higher odds to deliver at home compared with living in a community with a higher level of ANC utilization (Tables 4 and 5).

## Discussion

In Ethiopia, about nine in every ten births are delivered at home. This high proportion of home delivery is accounted for different individual and community-level characteristics, which affect women’s decision to deliver at home. At the individual-level, lower status of women and partners’ education, non exposure to mass media, making no or only a limited number of ANC visits, higher parity, and inaccessibility of health facility in terms of distance are positively associated with home delivery. At the community-level, women living in communities in pastoralist regions, rural areas, communities with higher poverty levels, those with lower ANC utilization, and communities where distance to a health facility is big problem are more likely to give birth at home.

Lower educational levels of mothers and their partners were positively associated with home delivery. This finding is consistent with different studies undertaken in Ethiopia [20–23], as well as in other countries [24–27]. Although representing only a slight effect, the decrease in proportion of home delivery in Ethiopia from 2005 to 2011 could be explained by the increasing possibility of education in the country [3], through community mobilization and expansion of schools. Education could influence women’s overall empowerment enhancing their ability to have self determination, access to information, and financial freedom to support themselves to take transport to a health facility and pay for (if applicable) for services, as well as to easily absorb health messages through the media and from health professionals. These could collectively influence mothers’ awareness to seek better medical services, including delivering in health facilities.

This study found that non-exposure to television and radio is associated with mothers giving birth at home, while exposure to media is associated with institutional delivery. Even a limited exposure to either radio or television has a positive effect on institutional delivery. This finding is consistent with other studies in Ethiopia, Pakistan, Indonesia, and India [28–30]. The effect could be explained by the fact that most media programs broadcast promotion of institutional delivery repeatedly that may influence mothers to develop positive behavior towards delivering in a health facility. However, in Ethiopia, low levels of electricity distribution in rural areas, where nearly 85% of the population lives in, and low educational levels could combine to reduce exposure to the media and thus women could fail to get the advantages of health promotions through media.

Antenatal care is a proximate predictor of women’s decision on place of delivery. That is, women who have had no prenatal care in a health facility are more likely to give birth at home. Even a limited number of ANC visits increase facility based delivery. Especially, making the minimum number of ANC visits recommended by WHO makes it more likely for mothers to give birth in a health facility [20–22,24,26,28,32]. As reported in DHS analysis of six African countries and in India, mothers who seek care for their pregnancy are more likely to seek care for their delivery [25,33]. Antenatal care is the most favorable contact point for mothers to get more information about risks and problems they may encounter during delivery. Consistent findings are reported in Tanzania and Rwanda studies in which mothers who are informed about pregnancy complications during their ANC visits are more likely to deliver in a health facility [34].

Individual and community perceived distance to health facility are associated with higher odds of home delivery. This seems to agree with the actual situation in Ethiopia. The most decentralized health facilities to the community are health posts, which serve nearly 5,000 people within their catchment areas and are staffed with health extension workers—community health workers with one year of training. However, these health facilities are not eligible to render delivery service; rather, they focus only on community mobilization, health promotion, and preventive care, including prenatal care. The next nearest structures that offer delivery service are health centers. But, most of the health centers are not accessible within a short distance, especially in the rural areas. With little transport availability to travel 10s and 20s kilometers, mountainous topography, women facing labor pains would find institutional delivery to be very challenging and would be likely stay at home for delivery. In addition, a low level ANC utilization and poor birth preparedness would lead women to have home delivery [26,32,35–37].

We also found that characteristics of clusters in which the women live have an effect on women’s decision on place of delivery, independent to their individual characteristics. For instance, living in a different contextual region makes a difference as to place of delivery. The 11 regions of Ethiopia, which are basically delineated for administrative purposes, were categorized into three contextual regions—pastoralist, agrarian, and city defined based on the basis of the living conditions of their population. As such, living in a pastoralist community is associated with higher chances of home birth, followed by an agrarian community. Home delivery is to be expected in a pastoralist community because pastoralist people are very hard to reach and mostly wander to distant areas to look for animal foods, in addition to having poor infrastructure. Generally, while the disparity in home delivery across the regions could be attributable to the differences in access to health services, media and information, geography, and social and cultural attributes, most important difference is the living conditions of their population. The reliability of this finding is assured by its consistency with previous studies conducted in Ethiopia and other African countries [24,28,31,36,38].

As expected, unlike urban communities, rural communities are at high risk of having home births, which is similar to findings in other studies [20,21,27,32]. The nature of urban and rural areas explains this discrepancy. Urban areas are accessible to health facilities, with a higher proportion of informed and educated people, and better infrastructure. In contrast, rural areas generally have poor infrastructure, hold deeply rooted negative beliefs and myths regarding institutional delivery, and are physically inaccessible and fewer health facilities, and inadequate health services. Collectively, the better situations of urban communities enhance health-seeking behavior of the population in general, not only for institutional delivery.

Regardless of the wealth status of individual women, poverty status of the cluster in which the women live determines the decisions of women regarding place of delivery. Relatively poor clusters are associated with a higher proportion of home delivery, and this finding is similar to other studies [24]. In addition, women’s decision to give birth at home is positively associated with clusters that have lower levels of ANC utilization. Not only does ANC utilization have a positive effect on their use of health facility for delivery, but also a higher community level of ANC utilization may influence women in the same community to give birth in a health facility. If a cluster has a high proportion of women who go for ANC, the information they receive during their ANC visits is likely to reach their neighbors and influence other women to seek medical services, including institutional delivery.

In sum, the result show the proportion of women who delivers at home is high in Ethiopia. This is accountable not only to the individual but also community-level factors. The study has implications for policy makers and programmers. First, it used robust methodology to analyze the data and it considered community-level characteristics associated with women’s decision on place of delivery. In addition, EDHS data is the most representative evidences of the nation. Policy makers have to design strategies that target to improve each of the individual women and community-level factors to reduce home delivery.
